# The Morals of the Workshop

**Published:** 1888-03-17

**Authors:** 


### The Doctor's Pulpit.

### THE MORALS OF THE WORKSHOP.

In a certain famous novel a village carpenter's shop, with its occupants, is described at some length and with much fidelity. Among other articles on which the men are engaged, a coffin is in course of construction. It is well known that in small country places a coffin is very seldom in request, and when such an article is really needed it is generally wanted in a hurry. The particular coffin of which we speak had for its constructor one "Wiry Ben." Now, Ben was by no means a bad fellow, but he had certain peculiarities which made him in George Eliot's eyes a proper subject for the pillory, and accordingly she pilloried him effectually for at least several generations. The coffin is wanted at once; Ben has got it completed, all except the driving in of the last nail. The nail is put in its place with a preliminary "tap," and only wants one ringing blow to drive it home. Ben lifts his hammer high in the air to give the blow, but suddenly, without driving the nail home, he lowers his upraised arm, drops the hammer into its place on the bench, and, putting on his coat, goes swinging out of the shop. What has happened to prevent the completion of the work which a single blow, occupying but the sixtieth part of a minute, would have brought to a termination? The first stroke of six has been struck by the village clock!

George Eliot, who probably depicted from life, considered an incident of that kind worth recording. Probably hundreds of workmen who read this page are familiar with men of the "Wiry Ben" type, and some may even claim kinship with Ben, and not be ashamed of it. It demands a moment's reflection why a woman so considerably removed by education and sympathy from the carpenter class should have thought it worth while to record and to carefully elaborate an incident of such an apparently trivial and altogether commonplace kind. But was it trivial? And what is triviality? If the driving of a nail is trivial, then the making of a coffin is trivial; and if the making of a coffin is trivial, the decent burial of the dead is trivial. Is a penny a trivial coin? Then twelve, or twelve hundred, or twelve million pennies may, by a simple process of reasoning, be proved trivial also. Is the title "carpenter" trivial? The logic which proves it so will, with tenfold force, prove—at least in these days—the title of "Duke" a thousand times more trivial still. The truth is, that no useful work is in its own nature small and insignificant, and no man who honestly earns wages by indispensable labour ought to consider himself, or be considered by others, as a person of no account.

In large populations individuals become relatively less important. Like the proverbial needle in the haystack, they are more easily lost than found. Some persons, by their inherited rank or wealth, stand out prominently, whatever be their character or ability, because the number of such can never be anything but small. But he who is born to carpentering or the making of shoes, though he may be quite as necessary, or even more necessary, to the prosperity of the commonwealth than the other, can only be, like an individual pea in a sackful, one among millions of others exactly like himself. Under the circumstances, it is natural and inevitable that he be overlooked almost entirely by the great and busy world. Such overlooking we find no fault with at all; it cannot be avoided. What we do find fault with, and what it is the object of this paper to point out and correct, is that the carpenter or the printer is in danger of *overlooking himself.*

It is this overlooking of a man's individuality and significance by himself which constitutes one of the chief dangers of modern times. A human being who has no marked individuality, no power of isolating himself so as to sometimes feel that he is alone in the world—alone with the summer and the winter, alone with birth and death, alone with his work and his strife among his fellows, alone with nature and with God—he who never feels this, can never be a man in the fullest sense of the word. He who oftenest feels it performs the tasks of life best of all men.

The large workshops and factories of the present day; the combination of men in guilds, associations, and clubs (against which we have not one word to say); the constant search for amusement and excitement when work is done; the crowding together of vast populations into small areas, narrow streets, small houses, and over-tenanted rooms—all these tend to prevent men realising their own individuality and their own significance in the world; and with the diminution of individuality there is a strictly corresponding and inevitable lessening of responsibility; and with the lessened responsibility there is a commensurate decline in efficiency of all kinds. These things are as certain as logic and every-day experience can make them.

We do not call attention to this question for the sake of vaguely deploring it, nor yet for the purpose of appealing to statesmen, patriots, and philosophers to devise means for counteracting its evils. For our part, we have small faith in what statesmen, patriots, or philosophers can do for their fellow-men. If a man desires to be at the other side of a stream over which there is no bridge, it is very seldom that he can with reason call upon any other man to carry him across. As a rule, the right and natural thing is for him to wade or swim, or otherwise get himself across in the best way he can. One of the most read of modern writers, by workmen, is Samuel Smiles, whose treatise on "Self-Help" is a classic in every cottage. Self-help is better than a gold mine, better than an inherited title, a fortune in the funds, a rich wife, or a perpetual pension.

The author of "Adam Bede" believed that a good many workmen had very feeble consciences, and only worked well when a master's eye was upon them. Many people as well as George Eliot affirm the same thing. They say that workmen are no better than big schoolboys; that men of forty or fifty, or even sixty, will do exactly what they must do, and no more; and that, in order to get them to do their work really well, it is necessary for somebody to look over them as constantly and as sharply as if they were children or slaves. Can this be true? If it be, every workman who is over thirty years of age ought to blush until his ears tingle with shame.

A haystack, even if large enough to weigh fifty thousand tons, consists of individual blades of grass and single leaves of herbs. The whole stack may be removed by taking away the single leaves and blades *one by one*. When they were growing each blade had a life of its own, a structure of its own; each leaf drew nourishment from the ground for itself, and for itself drank in the rain and dew. It is exactly the same with workmen. No matter how large the workshop, or how many thousands swing hammers, drive planes, or set types within its walls, each man and youth and boy is still a separate individual, who eats for himself, sleeps for himself, wears clothes for himself, takes wages for himself, and must work for himself. How should he work? There is only one way of doing work properly, and that is, always doing it as well as it possibly can be done. If a workman were suddenly turned into an employer, how would he like the work done? Perfectly, if possible, and no otherwise. Here, as elsewhere, the highest morality is the soundest policy; the tenderest conscience is the wisest guide; the noblest religion is the purest conduct. "Whatsoever ye would that men should do unto you, do ye even so unto them."

